# Anticancer nanoparticulate polymer‐drug conjugate

**DOI:** 10.1002/btm2.10033

**Published:** 2016-10-28

**Authors:** Quanyou Feng, Rong Tong

**Affiliations:** ^1^ Dept. of Chemical Engineering Virginia Polytechnic Institute and State University 635 Prices Fork Road Blacksburg VA 24061

**Keywords:** drug delivery, nanomedicine, nanoparticle, polymer‐drug conjugate, stimuli‐responsive

## Abstract

We review recent progress in polymer‐drug conjugate for cancer nanomedicine. Polymer‐drug conjugates, including the nanoparticle prepared from these conjugates, are designed to release drug in tumor tissues or cells in order to improve drugs’ therapeutic efficacy. We summarize general design principles for the polymer‐drug conjugate, including the synthetic strategies, the design of the chemical linkers between the drug and polymer in the conjugate, and the in vivo drug delivery barriers for polymer‐drug conjugates. Several new strategies, such as the synthesis of polymer‐drug conjugates and supramolecular‐drug conjugates, the use of stimulus‐responsive delivery, and triggering the change of the nanoparticle physiochemical properties to over delivery barriers, are also highlighted.

## 1 Introduction

In this review we feature various polymer‐drug conjugates (PDCs) based nanoparticles (NPs) used to deliver chemotherapeutics. Most of them are designed and fabricated to release drugs in tumor tissues or cells upon the triggering by different stimuli, in order to lower parent drugs’ systemic toxicities and improve their therapeutic efficacies.^1^ We illustrate some important lessons gleaned from over 60‐year development of PDCs, and discuss the promise and outstanding challenges facing the development of PDCs from a perspective of chemistry and materials engineering.

### 1.1 Polymeric nanomedicine for cancer therapy

Nanomedicine refers to the application of nanotechnology for the prognosis, diagnosis, prevention, and treatment of clinical conditions.^2^ Nanomedicine can enhance therapeutics and diagnostics in many ways, as has been reviewed.^3^, ^4^, ^5^, ^6^, ^7^, ^8^, ^9^ In cancer chemotherapy the NP enables the preferential delivery of drugs to tumors owing to the enhanced permeability and retention (EPR) effect—NPs are preferentially taken up by the leakier vasculature in tumor beds than small molecules and are retained because of the tortuous lymphatics.^10^, ^11^, ^12^ Several nanoparticulate therapeutics, for example, Doxil^TM^ (∼100 nm PEGylated liposome loaded with doxorubicin)^13^, Abraxane^TM^ (∼130 nm paclitaxel albumin‐stabilized NPs)^14^, ^15^ and Onivyde^TM^ (nanoliposome loaded with irinotecan),^16^ have been approved for use by the FDA, and have shown improved pharmacokinetics and reduced adverse effects compared to their parent drugs. Polymeric drug delivery NPs, one of the major delivery platforms, has actively evolved its paradigm from water‐soluble polymeric carriers, to liposome, micelle, dendrimer, polymersome, and other polymeric nanostructures.^17^, ^18^, ^19^

### 1.2 The development of the PDC

**Figure 1:**
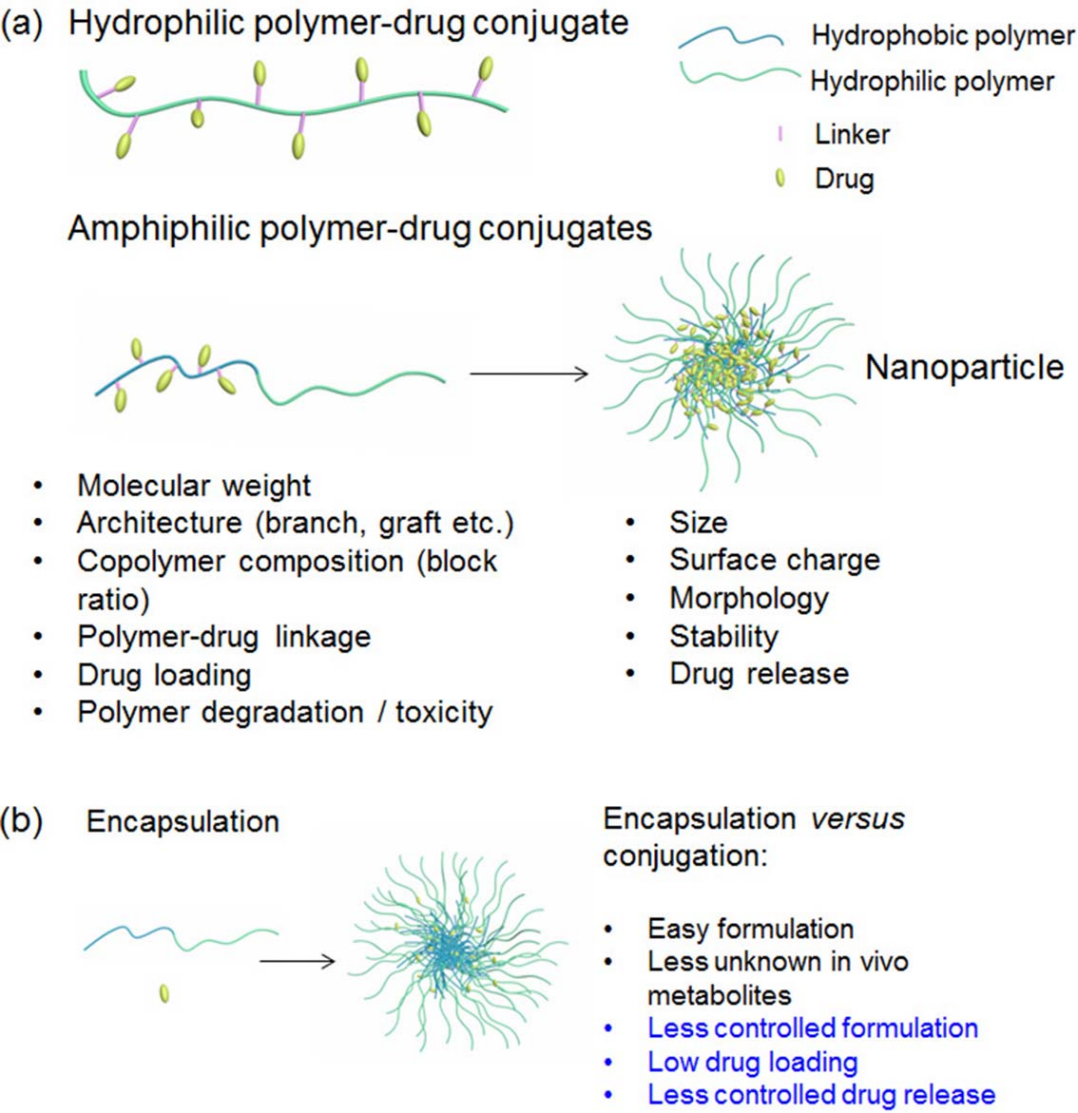
(a) Two representative polymer‐drug conjugates (PDCs): hydrophilic polymer‐drug conjugates, and nanoparticles composed of amphiphilic polymer‐drug conjugates. Both the polymer's and nanoparticle's physicochemical properties have to be well characterized for the future translation of PDCs. (b) Scheme of the nanoparticle encapsulating drugs, which is compared to the conjugation strategy

**Figure 2:**
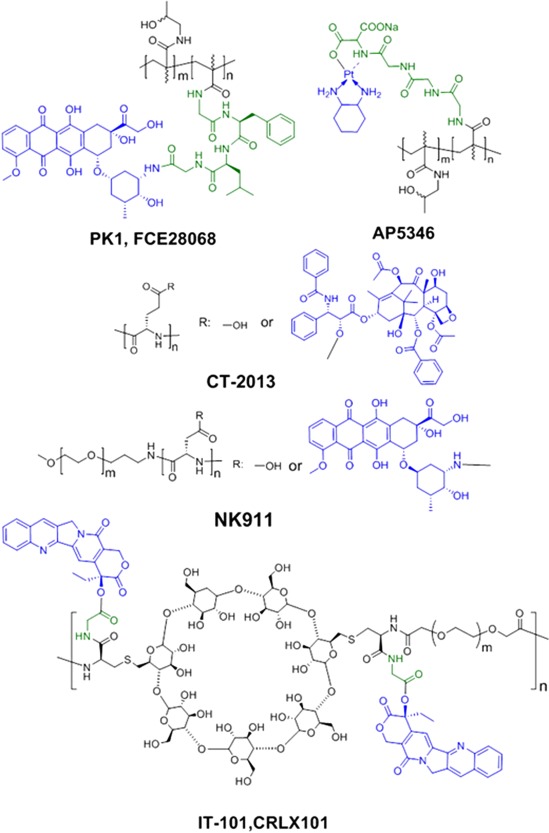
Chemical structures of some polymer‐drug conjugates in the clinical trials. The drugs are highlighted in blue, the linkers in green

PDC is one of the most important and oldest polymeric delivery systems (Figure 1). The conjugation of drugs to macromolecules was initiated about sixty years ago.^20^ Early work in 1950–1960s focused on numerous water‐soluble PDCs, especially poly(vinylpyrrolidone) conjugates.^21^ Mathé et al. pioneered conjugation of drugs to immunoglobulins in 1958, setting the stage for PDCs.^22^ In 1975 Ringsdorf presented a clear concept of the use of polymers as targetable drug carriers,^23^ which motivates rational design of the first generation of polymer therapeutics candidates (and first‐generation PDCs) that later entered clinical testing.^24^ Meanwhile Davies and coworkers modified proteins with poly(ethylene glycol) (PEG) to improve protein's circulation half‐life, immunogenicity, and stability,^25^ which leads to the development of therapeutic polymer‐protein conjugates. Of note, many of PEGylated protein conjugates have been approved for clinical use (e.g., Oncaspar^TM^, PEG‐L‐asparaginase, for treating leukemia), and will not be discussed in this review.^26^, ^27^ The important first generation PDCs include: poly(*N*‐hydroxypropyl methacrylamide) (polyHPMA), which is synthesized by Ulbrich and Kopeček, and later co‐developed with Duncan^28^, ^29^, ^30^, ^31^, ^32^, ^33^; poly(glutamic acid) with paclitaxel (Xyotax^TM^ or Opaxio^TM^) or camptothecin (CT‐2106) conjugates by Li and Wallace^34^, ^35^, ^36^; poly(styrene‐maleic anhydride)‐neocarzinostatin conjugate (SMANCS, Zinostatin Stimalmer^TM^) by Maeda, which is approved in Japan for the treatment of hepatocellular carcinoma.^37^, ^38^ In the late 1980s and early 1990s nanoparticulate drug delivery systems, including PEGylated polymeric micelles and liposomes, were rapidly developed after the discovery of EPR effect.^10^ Nanoparticulate form of PDCs in clinical trials also reached the clinic, including: CRLX101 (IT‐101) by Davis, a PEG‐cyclodextrin‐camptothecin polymeric micelle with 30–40 nm size^39^, ^40^; NK‐012, NK‐911 and NC‐6004 all developed by Kataoka, a PEG‐polypeptide block copolymer conjugated with SN‐38, doxorubicin or cisplatin, respectively (Table 1, Figure 2).^41^, ^42^, ^43^, ^44^, ^45^

**Table 1:** Representative polymer‐drug conjugates in clinical trials

Name	Brand name	Polymer composition	Drug	Linker	Status	Molecular weight (kDa)	Loading (wt%)	Size (nm)	Plasma half‐life (h)	AUC (h·mg/L)	Cmax (mg/L)	Other	References
PK1, FCE28068		HPMA copolymer	Doxo	GFLG peptide	Phase II (unknown)	30	8.5	7.8	93	N.S.	65	1.3% 24 hrs in head‐neck tumor; 50‐75% dose undergo renal clearance; 6/62 patients showing partial response	32, 33, 75
PK2, FCE28069		HPMA copolymer	Doxo/galactosamine	GFLG peptide	Phase I (completed); Phase II (unknown)	25	7	10.5	28	296	N.S.	16.9% 24 hrs in liver for hepatic tumor, but only 3.3% in the cancerous regions of the liver	31, 75
AP5346	ProLindac	HPMA copolymer	DACH‐Pt	GGG‐carboxylate‐Pt coordination	Phase II (unknown)	25	10	N.S.	72.3	136	13		59
PCNU1661 48		HPMA copolymer	Cpt	Ester	Phase I (stopped)	18	10	N.S.	N.S.	N.S.	N.S.		129
CT‐2103	Xyotax, Opaxio	Poly(glutamic acid)	Ptxl	ester	Phase II (completed); Phase III (ongoing)	39	36	N.S.	120	1583	N.S.	2/26 NSCLC patients showing partial response; 9/44 patients showing partial response in the combination with cisplatin; 4/12 having complete response in gastric and esophageal cancers in combination with radiation	34, 35, 263
CT‐2106		Poly(glutamic aid)	Cpt	Ester	Phase I (completed)	49	37	N.S.	51	36	14		36
EZ‐246	Pegamotecan	PEG	Cpt	Ester	Phase II (terminated)	40	2	N.S.	46	27	0.5		57
AD‐70		Dextran	Doxo	Imine	Phase I (completed)	70	N.S.	N.S.	11	N.S.	0.01		70, 71
NK911		PEG‐*b*)‐poly(aspartic acid)	Doxo	Amide	Phase II (unknown)	16	17	40	7.5	3.2	3.9		41, 42
NK012		PEG‐*b*)‐poly(glutamic acid)	SN‐38	Ester	Phase II (completed)	19	20	20	137	294	19.1		42, 43
NC6004	Nanoplatin	PEG‐*b*‐poly(glutamic acid)	Cisplatin	Pt‐carboxylate coordination	Phase III (recruiting)	26	30	30	129	2836	60.8		42, 44, 45
NC6300		PEG‐*b*‐poly(aspartic acid)	Epirubicin	hydrazine	Phase I (unknown)	20	20	60	N.S.	N.S.	N.S.		42
NC4016		PEG‐*b*)‐poly(glutamic acid)	DACH‐Pt	Pt‐carboxylate coordination	Phase I (recruiting)	26	30	30	N.S.	N.S.	N.S.		42
CRLX101, IT‐101		poly(cyclodextrin)‐*co*‐PEG	Cpt	glycine	Phase I (completed); Phase II (recruiting)	57	6.8	36	27.9	306	8.3	3/19 patients show partial response; 14/19 having net tumor reduction	39, 40

DACH = 1,2‐diaminocyclohexane; Doxo = doxorubicin; Ptxl = paclitaxel; Cpt = camptothecin; HPMA = *N*‐(2‐hydroxypropyl) methacrylamide; N.S. = not stated; AUC = the area under the plasma concentration‐time curve; *C*_max_ = maximum drug concentration.

### 1.3 Stimuli‐sensitive PDC

Although it is suggested that the EPR effect exist in human tumors,^46^, ^47^ it is still questionable whether the EPR effect is sufficient to significantly improve the survival of cancer patients by nanomedicine.^48^, ^49^ Several delivery barriers limit the transport of NPs deep into tumors^4^, ^50^; (see Section 2.4) recent advances in biology show that abnormal tumor microenvironments help tumor progress and resist the treatment.^51^ Therefore the stimuli‐responsive NPs are designed to overcome the delivery barriers in tumor microenvironment to improve the therapeutic efficacy.^52^ In fact many PDCs contain stimuli‐sensitive linkers positioned between the drug molecule and the polymer; the drug remains on the PDC in circulation and can be locally triggered release, by either endogenous stimuli in tumor microenvironment such as pH or enzyme, or by applied endogenous stimuli on tumor, such as light or heat source (see detailed discussion in Sections 2.3, 3.3–3.5).

### 1.4 Loading drugs in NPs: encapsulation versus conjugation

The method of drug loading imposes numerous design constraints on the delivery platform. The drug‐encapsulated delivery platform has continuous drug release during the circulation, which making it difficult to achieve therapeutically effective concentration at tumor and could cause systemic side effects in normal tissues (Figure 1b).^53^, ^54^ The covalent linkage between the drug and polymer in the PDC offers opportunities for triggered release only at the tumor tissue or cell. In addition, a high drug loading can be achieved relatively easily in PDCs compared with drug‐encapsulated NPs. Higher drug loading of delivery vehicles is desirable for optimal therapeutic effect, to enhance the potency of NPs that reach the tumors.^55^ However, one obvious shortcoming for PDCs is that not all of the drugs have chemical functional groups for covalent conjugation. Fortunately, many prevalent chemotherapeutics, including paclitaxel (Ptxl), docetaxel (Dtxl), doxorubicin (Doxo), gemcitabine, irinotecan, and camptothecin (Cpt), can be conjugated to polymers. In addition, PDCs may require tremendous synthetic efforts compared with encapsulation. Furthermore, the in vivo characterization of PDC's stability, release, metabolism, excretion and toxicity can be demanding: a PDC is viewed as a new drug by FDA, and its’ metabolites’ toxicity and pharmacokinetics require detailed examination.^55^

Overcoming these challenges requires (a) the judicious chemistry design to ensure tumor‐specific drug release; and (b) qualitative preclinical in vivo characterization of PDCs’ pharmacokinetics, drug release and metabolism for better understanding. Here we mainly focus on the stimuli‐sensitive PDCs in this review. We first summarize some design principles of PDCs based on the preclinical studies (Section 2), including the polymer and conjugation linker's chemistry, NP's physicochemical properties, and the in vivo delivery barriers requiring design consideration. We then highlight recent strategies in the development of PDCs (Section 3), aiming to address challenges in chemistry, materials and in vivo application of PDCs.

## 2 The design principles of PDC for cancer nanomedicine

There are a number of overarching designing principles in the delivery of PDCs to tumor sites, which recur throughout this review. Most of these are based on the preclinical findings in animal models. Some of these are common to many other delivery carriers where NPs are of significant interest, while others are unique to the polymeric chemistry and materials in PDCs.

### 2.1 Polymer

Polymers that have functional groups for the incorporation and release of drugs in PDCs must be well characterized (Figure 1a). All in vivo metabolic products of PDCs should be nontoxic and nonantigenic. Polymers in PDCs should be either biodegradable or completely eliminated from the body. In this review, various polymers are discussed, including: hydrophilic polymers used in first‐generation PDCs for clinical trials, such as polyHPMA, and PEG^1^, ^56^, ^57^, ^58^, ^59^; copolymers, especially block copolymers that can be formulate to nanostructures such as micelle^60^ or polymersome^61^, ^62^; dendrimers^63^ and hyperbranched polymers,^64^, ^65^ and natural macromolecules such as polysaccharides (dextran, cellulose, chitosan) and polypeptides.^66^, ^67^, ^68^, ^69^, ^70^, ^71^

#### 2.1.1 Polymer molecular weight

The polymer molecular weight affects the in vivo circulation of hydrophilic polymers. In general the higher the molecular weight, the longer the intravascular half‐life and the slower the elimination of hydrophilic polymer based conjugates from the body. Such trend has been shown in the studies of polyHPMA,^72^, ^73^, ^74^, ^75^ dextran^76^, ^77^ and dendrimers, etc.^78^ The half‐life of polyHPMA‐Doxo conjugate (molecular weight 1230 kDa) in blood was up to 28 times longer, and the elimination rate from the tumor was 25 times slower than that of free Doxo.^72^

#### 2.1.2 Polymer architecture

Hydrophilic polymer architecture has an important impact on the in vivo activity of the PDCs. Ulbrich's group studied in detail the relationship between the architecture of HPMA copolymers—linear conjugates, branched conjugates, grafted conjugates, self‐assembled micellar conjugates, and grafted dendritic star conjugates—and their activity.^79^ Other studies showed the impact of the polymer architecture (conformation, flexibility, branching, and hydrodynamic volume) on the fate of the circulation of polymers in vivo. The polymer architecture has a serious impact on the clearance of polymers through the kidney.^80^ Large‐sized hydrophilic polymers with decreased flexibility, and an increased number of polymer chain ends, help prevent elimination of the polymer by the kidneys and can improve blood circulation time. However, the polymer architecture has much smaller effect on the extravasation of the polymer into the tumor.^80^

#### 2.1.3 Block copolymer's composition

The relative ratio of the hydrophobic to hydrophilic block length profoundly affects the NP's morphology.^81^, ^82^, ^83^ Typically the morphology of prepared amphiphilic block copolymer NP is spherical, particularly if the molecular weight of the hydrophilic block exceeds that of the hydrophobic block (so‐called star micelles). However, if the copolymer is asymmetric in its relative block lengths (i.e., the hydrophobic block is considerably longer than the hydrophilic block) during the self‐assembly process, varying morphologies can be obtained.^84^, ^85^ In addition, the copolymer's concentration in water‐miscible solvent affects the final NPs’ size.^86^ The use of triblock polymers could improve the NP's stability.^54^ Nevertheless, there lacks systemic studies on the block copolymer ratio or composition on the in vivo circulation and stability of the NPs’ morphology, presumably due to the technical difficulty to monitor the sub‐100 nm polymeric NPs in vivo. Recent in vivo pharmacokinetic studies using dual‐radiolabeling of lipid and drug in liposomes could provide a valuable example for the study the biodistribution of copolymer‐based PDCs in vivo.^87^

Notably, recent studies have shown that the zwitterionic copolymers (i.e., polymers containing both cationic and anionic groups) are super‐hydrophilic, which can prolong the circulation of NPs in the way similar to PEG.^88^

### 2.2 Synthetic strategy of PDC

**Figure 3:**
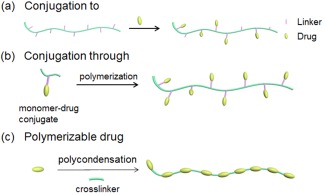
Three synthetic strategies of polymer‐drug conjugates

There are three strategies for drug‐polymer conjugates (Figure 3).^89^ The first is conjugating a drug to a pre‐synthesized polymer, named as “conjugation to.” The second is to conjugate a drug to a monomer prior to polymerization, namely “conjugation through.” The last is the polymerization of drugs to prepare PDCs, where drugs are directly used in the polymerization as monomers or initiator.^90^ The last two strategies have been recently developed to prepare PDCs, aiming to overcome the non‐controlled drug conjugation problem in the “conjugation to” strategy (see Sections 3.1 and 3.2).

### 2.3 Drug release and the conjugation linker

An ideal PDC for cancer treatment should be able to release the drug in tumor tissues or cells, but not to the normal tissues or cells. Two types of linker can be positioned between drug and polymer: cleavable linker and non‐cleavable linker. Non‐cleavable linkers, such as thioether linkers, have been seen in the antibody‐drug conjugates (ADCs).^91^ The release of the drug from these ADCs requires complete hydrolysis of the polypeptide backbone of the antibody in cell lysosomes.^92^ One example is T‐DM1 (Kadcyla^TM^), an ADC to treat metastatic breast cancer, which has the thioether linker, and exhibited better antitumor efficacy than the same ADC but with disulfide linker.^93^ However, the use of non‐cleavable linker of degrading the delivery platform after cell uptake might not be feasible in more complex NP systems.

**Figure 4:**
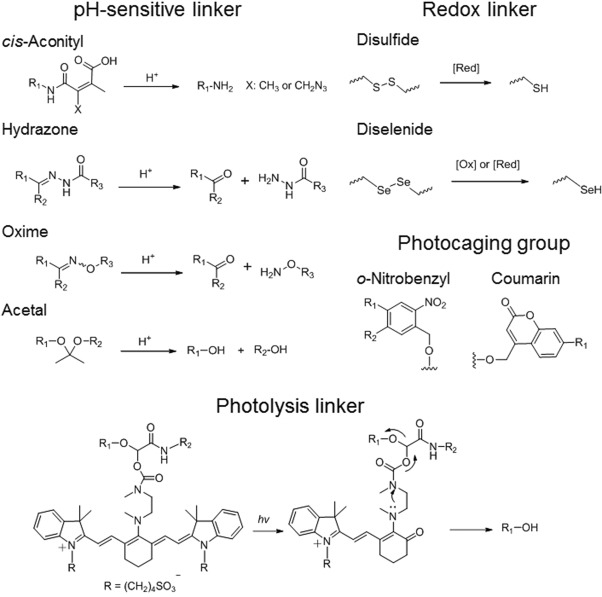
Schemes of various stimuli‐responsive linkers used in polymer‐drug conjugates, including pH‐sensitive linkers, redox‐sensitive linkers, photocaging groups, and photolysis linkers

Enormous synthetic efforts have been devoted to design stimulus‐sensitive cleavable linkers to trigger drug release (Figure 4). During the NP's extravasation, local tumor microenvironmental factors, such as pH (6.7–7.0),^94^ redox state (hypoxic tumor microenvironment^95^ and elevated reactive oxygen species generated by tumor cells^96^) and specific molecules overexpressed in tumor (e.g., matrix metalloproteinases [MMP]),^97^ can be utilized to disrupt PDCs’ structures to release loaded drugs, or induce NPs size or morphology change for enhanced penetration (see Section 3.5). Besides the endogenous stimuli, the external stimuli—such as magnetic field, temperature, light, and ultrasound—can be applied in a spatiotemporal manner to control drug release.^98^, ^99^, ^100^, ^101^, ^102^

More importantly, cleavable linkers have to result in direct release of the drug from the remaining linker fragment upon the cleavage, that is, no prodrug released. For systemically delivered PDCs, these linkers should be stable in circulation to avoid the side effects from the free drug and/or the decreased drug accumulation in tumors. We mainly discuss each type of stimulus‐sensitive linker, focusing on the general chemistry, in vivo stability and some preclinical successful examples.

#### 2.3.1 pH sensitive linker

The mildly acidic pH in tumor tissues (pH ∼ 6.7–7.0)^94^ as well as in the endosomal intracellular compartments (pH ∼ 4.5–6.5)^103^, ^104^ can trigger drug release from pH‐sensitive PDCs upon their retention at tumor sites. Many pH‐sensitive PDCs have been developed including *cis*‐aconityl amide, hydrazone, imine, oxime, acetal/ketal/orthoester,^105^ or other groups like trityl, *N*‐ethoxybenzylimidazoles and thiopropionate,^106^ and silyl ether etc (Figure 4).^101^, ^105^, ^107^

For Doxo PDCs, the acid sensitive hydrazone linker is often used to conjugate polymers to the ketone group in Doxo. However, the acid‐labile hydrazone linker is relatively unstable in vivo, with half‐lives in plasma of 48–72 hrs, less than that of the antibody moiety.^108^ In some cases, some hydrazone linker could induce the cyclic reaction and release less active Doxo prodrug, instead of free Doxo.^109^ Some other Doxo conjugates containing pH‐sensitive cis‐aconityl spacer were prepared by the reaction of amino group of Doxo with *cis*‐aconitic anhydride forming α,β‐unsaturated amide.^110^, ^111^, ^112^, ^113^, ^114^

#### 2.3.2 Redox sensitive linker

The difference in redox potential between normal and tumor tissues, and between the intracellular and extracellular environment, can be exploited for triggered drug delivery.^115^ In the nanomedicine field, it is generally believed that the concentration of glutathione, a reducing tripeptide with thiol group, in cancer cells is 100‐ to 1,000‐fold higher than in the blood, and in a tumor mass the glutathione concentration is also markedly (100‐fold) higher than the extracellular level of glutathione in normal tissue.^116^ However, studies showed that in mice model a total fourfold higher level of glutathione in tumor tissues compared with normal tissues, and there exists significant heterogeneity of redox status in the tumor tissue.^117^ In human cancer patients, glutathione levels tend to be elevated in breast, ovarian, head and neck, and lung cancers compared with disease‐free peritumoral or healthy tissue; conversely, brain and liver tumors patients exhibit lower tissue level of glutathione in tumor compared with that in healthy tissue.^118^ In addition, two studies concluded that glutathione levels did not differ between parenchymal tissue sampled from healthy patients and uninvolved parenchymal tissue from lungs with tumors.^119^, ^120^ Therefore, right preclinical models and tumor types have to be rationally chosen when applying redox‐sensitive PDCs.

The reducing materials in vivo could facilitate the cleavage of redox‐sensitive bonds such as disulfide bond and diselenide bond.^121^ For example Kopeček and coworkers conjugated the photosensitizer mesochlorin e6 to HPMA copolymer via a disulfide bond, which showed a time‐dependent release of Mce6 and concomitant increase in the photodynamic efficacy when exposing to DTT.^122^ However, the disulfide‐based linker showed relatively short in vivo stability less than 1 day in ADCs, respectively, which is much shorter than the parent antibody moieties.^108^ The in vivo circulation stability PDCs containing redox‐sensitive linker should be evaluated carefully in future.

#### 2.3.3 Enzyme sensitive linker

The increased expression of certain local enzymes in cancer, such as MMP, not only can be regarded as a biomarker for disease diagnosis and prognosis, but also represents a means for enzyme‐triggered drug release in tumor.^123^, ^124^, ^125^, ^126^ Early studies on detailed degradation studies of oligopeptide sequences attached to polyHPMA‐based PDCs identified the short peptide GFLG, specific for cathepsin B.^127^ The polyHPMA PDCs with such linker have shown efficacy in various preclinical efficacy study and have entered the clinical trials.^128^, ^129^ Another widely used short peptide linker is citrulline‐valine, which can be cleaved by specific lysosomal proteases but impart greater stability in plasma. Of note Brentuximab vedotin (Adcetris^TM^ approved for the use in Hodgkin lymphoma) is an ADC containing such dipeptide linker to facilitate release of the drug, monomethylauristatin E.^130^ Other short peptides linkers include PVGLIG (cleaved by MMP‐2/MMP‐9),^131^ and SSKYQL (cleaved by prostate‐specific antigen).^132^ One can envision that the presence of certain enzymes as biomarkers potentially could be utilized to design a PDC for personalized medicine, once the concentration of enzymes at the tumor site should also be sufficient for the disruption of the PDC.

#### 2.3.4 Light sensitive linker

There has recently been growing interest in light‐responsive NPs for triggered drug delivery. The use of an optical stimulus is appealing because it could provide a greater selectivity in terms of control over the moment and the location of drug release, and potentially transfer photonic energy to heat, acoustic wave, or induce reactive species such as singlet oxygen in photodynamic therapy.^133^ In terms of light‐triggered chemical bond cleavage, several classes of light‐sensitive linkers have been reported including the nitrobenzyl, coumarin‐4‐yl‐methyl, *p*‐hydroxyphenacyl, and 7‐nitroindoline derivatives with ester, amide, carbonate, carbamate, and phosphate linkages for photolysis (Figure 4).^134^ However, many photocaging groups required the irradiation by UV light or short wavelength visible light, which restricted primarily to superficial lesions unless fiberoptics or near‐infrared (NIR) light can be used. Of note, NIR light, with wavelengths in the range of about 700–1000 nm, is more suitable for biomedical applications than UV or visible light; the irradiation is less detrimental to healthy cells, and the absorption and scattering by water and biological substances are reduced, which results in a greater tissue penetration depth (on the order of millimeters to centimeters).^135^, ^136^, ^137^

One way to use NIR light is to use two‐photon excitation for many UV‐light absorbing photosensitive linkers.^138^, ^139^ Two‐photon excitation usually requires high‐intensity pulsed laser (MW·cm^−2^ to GW·cm^−2^)^140^; however, many photocaging groups do not have large enough two‐photon cross‐sections to be efficiently activated by NIR light.^141^ Alternatively, recent developed NIR‐light sensitive linkers are showing promise. A near‐IR (690 nm) light‐initiated photolysis reaction was developed based on the C4’‐dialkylamine‐substituted heptamethine cyanine linker and has been used in a light‐triggerable ADC; upon the irradiation, the photo‐oxidation of the cyanine polyene could generate a secondary amine and promote the cleavage of the carbamate bond to release the drug (Figure 4).^142^, ^143^ The use of inorganic NPs such as gold NPs or upconversion NPs for triggered drug release offers another intriguing strategy and has been reviewed elsewhere.^144^, ^145^, ^146^

### 2.4 In vivo drug delivery barriers for PDCs

#### 2.4.1 Circulation

To achieve therapeutic efficacy, NPs must first overcome systemic barriers with prolong circulation time, especially clearance by mononuclear phagocytic system (or so‐called reticuloendothelial system), hepatobiliary system and urinary system.^147^ In general, NPs with sizes below 100 nm are suitable for systemic (usually intravenous) distribution, as larger ones cause embolic phenomena^148^; while there seems no significant difference in circulation half‐life for various sized NPs in the range of 30–100 nm.^149^ To avoid rapid clearance by the kidneys, the NP's hydrodynamic size should be larger than 6 nm.^150^ Notably, most HPMA PDCs in clinical trials have NPs size below 10 nm (Table 1) or moderate molecular weight (less than 40 kDa, Table 1), which may affect their in vivo circulation and accumulation profiles. The NP should keep its size in circulation and not be destabilized under flow or at physiological temperature.^151^ In addition, the NP should not bind with proteins in blood that could lead to aggregation, or be uptaken by the macrophages in the mononuclear phagocytic system, all of which can lower the dose of NPs reaching tumors.^152^ Coating of NPs with PEG that mimics a cell's glococalyx,^153^, ^154^, ^155^ known as “PEGylation,” can suppress protein absorption to NPs and delay the rate of NP uptake and clearance, greatly prolonging circulation time.^156^ The NP's circulation half‐life is impacted by the extent of PEGylation on NPs surface,^157^, ^158^ and may be reduced upon repetitive administration, which has been reviewed elsewhere.^159^, ^160^

#### 2.4.2 Tumor penetration

When NPs reach tumor blood stream from circulation NPs extravasate from tumor vessels and penetrate up to hundreds of micrometers through the tumor stroma so that even cancer cells situated distal to the tumor vessel can be exposed to the anticancer agent at high enough concentrations. Thus, both NPs accumulation (total mass) and penetration depth from vessels over the time can determine the efficacy and have to be carefully examined in preclinical studies, which can be evaluated by both the drug's concentration in tumor and the area under the drug's intratumoral concentration–time curve. NP size is one crucial determinant of accumulation and penetration into tumor tissue. It is reported that polymeric micelles ∼ 30 nm showed enhanced tissue penetration and potent anti‐tumor activity in pancreatic tumors, compared with larger NPs.^149^ In another example, 50 nm NPs showed deeper tissue penetration and higher accumulation in breast tumors over time, compared with 20 nm or larger NPs.^161^ One recent imaging study showed that the intercellular gaps and transcellular fenestrae in the tumor have dynamic changes that brief vigorous outward fluid flows into the tumor interstitial space, which allows for the 70 nm sized NPs extravasate into tumor tissues.^162^ In general, current consensus is that sub‐100 nm may be the optimal NP size range for passive tumor targeting, which may vary depending on individual NP's composition and formulation. Besides, the NP surface charge (see discussion in Section 3.5.2) and the aspect ratio of NPs can affect NP's, in vivo circulation time and tumor penetration capability^163^ and NP's cell uptake.^164^ Other strategies of improving NP's tumor penetration include co‐injecting drugs to reduce tumor's extracellular matrix density,^165^, ^166^ and conjugating tumor‐homing or tumor penetration ligands.^167^, ^168^

#### 2.4.3 Tumor cell uptake

After reaching the tumor cells, NPs may need to cross the barrier of the cell membrane to deliver the loaded drugs into specific organelles to achieve efficacy. The surface modification of NPs with cell targeting ligands,^169^ cell penetration peptides,^170^ or lysosome‐destabilizing agents^171^ can greatly enhance intracellular uptake. Generally cancer cells may contain certain receptors or targets, such as transferrin, EGFR/HER‐2, PSMA, VCAM, that can mediate the corresponding enhanced cellular uptake of targeted NPs.^172^ Of note, the use of targeting ligands can enhance NPs’ cellular uptake but not necessarily increase the tumor accumulation of NPs when compared with EPR‐mediated accumulation.^173^, ^174^, ^175^ Conversely, the introduction of targeting ligands onto NPs not only requests synthetic efforts but also sometimes compromises the prolonged circulation of PEGylated NPs.^176^, ^177^ The surface density of targeting ligands should be closely monitored to provide a desirable targeting effect without reducing NP's circulation or tumor penetration capability.

For NPs without targeting ligands, intracellular NPs are found mainly within endosomes or lysosomes. These organelles have acidic pH, and contain proteases for degradation. The rate of uptake and intracellular localization of NPs have been studied by many research groups.^178^, ^179^, ^180^ Currently it is difficult to draw general conclusions about optimal physicochemical properties of NPs for rapid cellular uptake, since the rate and mechanism of uptake are cell‐type dependent and could vary between NPs with different size, charge, and other surface properties. However, some reports show that NPs of 20–50 nm are taken up more rapidly than smaller or larger NPs.^178^, ^181^

For hydrophilic polymer based PDCs, it is found that some PDCs, such as polypeptides^182^ or dextran,^183^ cannot be naturally degraded into small fragments that can cross the lysosomal membrane; the accumulated polymers in the lysosome increase the osmotic pressure and adversely affect the biocompatibility.^184^ Another study shows that most polyHPMA‐based PDCs quickly and evenly diffuse throughout the cytoplasm and remain excluded from membrane‐bound organelles; only strongly cationic HPMA copolymers can bound to microtubules; the nuclear entry kinetics were affected by the ratio of the HPMA to comonomer compositions.^185^

## 3 New strategies in PDC

The purpose of this section is to highlight some novel ways in which chemistry and nanotechnology are being applied to tackle challenges in PDC development.

### 3.1 “Conjugation through” PDC

The “conjugation through” method in PDCs requires monomer‐drug conjugates not interfere the polymerization.^89^ The “conjugation through” method could address the drawbacks in the “conjugation to” strategy, such as inconsistent and uncontrolled site conjugation along the polymer backbone.^89^ The drug loading can be controlled by adjusting the feed ratio of monomer‐drug conjugates; and the drug release can be controlled by the judicious selection of the linker between the drug and the monomer, which could be stimulus‐responsive (Figure 3b).^186^ Such method thus allows for even higher drug‐loadings than the “conjugation to” approach, by avoiding steric hindrance and accessibility limitation during the conjugation.

A few of monomer‐drug conjugates have been synthesized to prepare PDCs and corresponding NPs. Ring‐opening metathesis polymerization (ROMP) is often unitized in the “conjugation through” strategy. Examples include the norbornene‐Doxo conjugate with the acid‐sensitive carbamate linker,^187^ or the similar norbornene‐Doxo conjugate with the hydrazone linker.^188^ Multiple drugs including Doxo, Cpt, and cisplatin were individually tethered to the norbornene monomer with different stimulus‐responsive linkers; upon ROMP, precise ratio of drugs were controlled linked to the polymer, and resulted NPs could orthogonally triggered release individual drugs.^189^, ^190^ Similarly, the reversible addition fragmentation transfer (RAFT) polymerization is reported for “conjugation through” method; for example, Cpt‐tethered acrylate with redox‐sensitive disulfide linker was polymerized by RAFT to formulate redox‐sensitive NPs.^191^ However, PDCs synthesized by the ROMP or RAFT had non‐biodegradable polymer backbones, which limits their potential clinical application.^186^ Alternatively, the ring‐opening polymerization (ROP) is applied in “conjugation through” method. For instance, a campothecin‐tethered cyclic carbonate monomer was prepared with the disulfide linker between the drug and carbonate. The ROP of such drug‐carbonate conjugates resulted in a biodegradable polycarbonate PDC which can be further formulated to redox‐responsive NPs.^186^

### 3.2 Polymerizable drug

The use of drug as the monomer could significantly increase the drug loading. However, not many drug molecules fit for such strategy. Often, drug molecules contain two functional groups that allow for polycondensation reaction. Another drawback lies in the polymerization chemistry especially in polycondensation reaction. Such polymerization cannot produce high molecular‐weight polymer (e.g., over 10 kDa) and lacks polydispersity control. In addition, the introduction of stimulus‐responsive group is not straightforward. Early work to prepare PDCs via such strategy often focus on the polyanhydrides which degrades through hydrolysis in vivo without burst release. Drugs such as ibuprofen, naproxen,^192^ ferulic acid,^193^ or morphine^194^ have been used as monomer in the polymerization.

**Figure 5:**
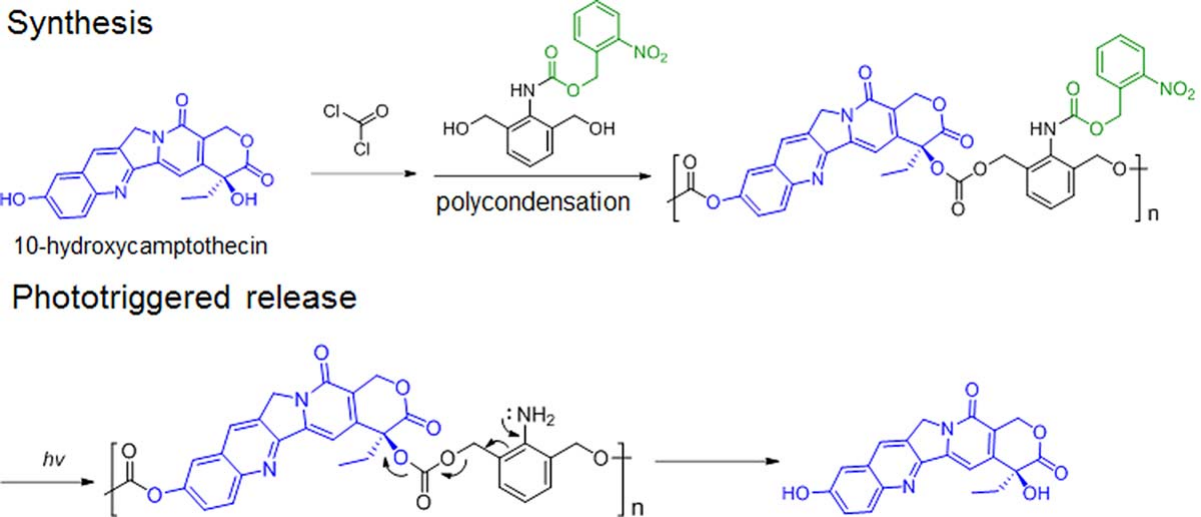
The synthesis and drug release of a light‐triggerable polymer‐10‐hydroxylcamptothecin conjugate. The drugs are highlighted in blue, the linkers in green

Recently a new facile strategy has been reported to use the stimulus responsive group to induce further depolymerization in the PDCs using drugs as monomers. 10‐hydroxycamptothecin, a diol drug, was polymerized with *o*‐nitrobenzyl, a photosensitive group, caged 2,6‐bis(hydroxymethyl)aniline via condensation polymerization. The resulted polycarbonate PDCs could be responsive to the UV‐light triggering: the *o*‐nitrobenzyl group was detached from the polymer and unfolded the aniline groups, which could successively trigger the depolymerization via the 1,4‐elimination reaction (Figure 5) and released the drug.^195^ Similar 10‐hydroxycamptothecin‐loaded polycarbonate caged with redox disulfide linker was also reported.^196^

### 3.3 Light‐responsive PDC

#### 3.3.1 Photosensitizer conjugate

Photodynamic therapy is a photochemistry‐based approach for treating tumors or other diseases such as macular degeneration. It involves the administration of nontoxic dyes known as photosensitizers systemically or topically, followed by illumination of the lesion with visible or NIR light,^197^ and then photosensitizers generate cytotoxic oxygen species (either singlet oxygen or oxygen radicals).^198^ Most photosensitizers bind to normal cells as well as to cancer cells, leading to unwanted off‐target activation from environmental (ambient) light.^199^, ^200^, ^201^, ^202^ The conjugation of photosensitizer to polymeric delivery vehicles is designed to improve photosensitizer's performance by increasing specificity and/or uptake in tumors, or decreasing phototoxicity to normal tissue.^203^, ^204^ Early photosensitizer‐drug conjugates include polyHPMA, PEG and antibody conjugates.^205^, ^206^, ^207^, ^208^ Factors such as the charge and hydrodynamic size of the conjugates affect the cellular uptake rate and tumor accumulation of hydrophilic polymer‐photosensitizer conjugates.^209^, ^210^ In many cases the covalent linkage between photosensitizer and polymer significantly reduced the quantum yield.^211^ The enzymatic‐cleavable linker, or environmental‐sensitive linker, was introduced to enhance both the selectivity of photosensitizer and the quantum yield; the conjugates were quenched and non‐toxic in the native state, but became fluorescent and produced singlet oxygen upon the cleavage of linkers by proteases in tumor.^211^, ^212^, ^213^, ^214^

The use of lipid‐photosensitizer conjugates to formulate light‐sensitive liposomes that combine photothermal therapy with chemotherapy has recently garnered interest. Photothermal therapy may potentially improve the chemotherapy efficacy of polymeric NPs containing drugs.^215^ For example, nanoliposomes composed of lipid conjugates of pyropheophorbide (a chlorin analogue) can efficiently absorb and transfer light energy into heat for photothermal therapy, as well as release the loaded drugs inside liposome.^216^ Of note, the use of another porphyrin‐lipid conjugates could also induce the transient increased permeability of the nanoliposome upon NIR light triggering; its mechanism remains unknown but not due to the photothermal effect.^217^

#### 3.3.2 Conjugated polymers

**Figure 6:**
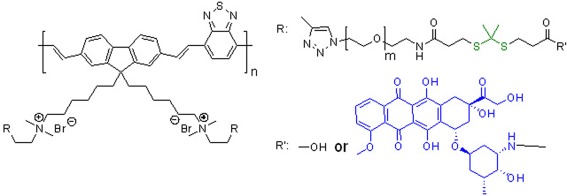
The chemical structure of a conjugated polymer‐doxorubicin (blue) conjugate with redox‐sensitive thioacetal linker (green)

Conjugated polymers, or conductive polymers, containing light‐absorbing units in their backbones with delocalized electrons (overlapping p‐orbitals), have attracted interests in applications ranging from light‐emitting diodes, photovoltaics to sensors.^218^, ^219^ Some conjugated polymers can generate ROS upon the irradiation of light, and become a new class of materials for photodynamic therapy.^220^, ^221^ A light‐sensitive PDC can be formulated using redox‐sensitive or ROS‐cleavable thioacetal linker, between the drug and the conjugated polymer; upon light illumination, the generated ROS causes drug release through the cleavage of the thioacetal linker (Figure 6).^222^ Such nanoparticulate PDCs can be triggered by visible or NIR light, providing new opportunities for both photodynamic therapy and chemotherapy delivery, as most light‐responsive polymeric systems are still activated by UV light.^133^

### 3.4 Thermal‐responsive PDCs

One of the most promising thermal‐responsive polymers used in PDCs is the elastin‐like polypeptide (ELP). ELPs are biopolymers with the pentapeptide repeating unit Val–Pro–Gly–Xaa–Gly, where Xaa can be any of the natural amino acids except Pro.^223^ Aqueous ELP solution undergoes an inverse temperature phase transition; the soluble solution becomes hydrophobically aggregation when heated up above its transition temperature, which can be adjusted ∼ 40–42°C for hyperthermia application.^224^ In such context, ELP‐drug conjugates have prolonged circulation with the half‐life over 8 hrs,^225^ and could accumulate in the locally heated tumor region, which was confirmed by intravital fluorescence microscopy.^226^ The use of acid‐ or redox‐sensitive linkers in ELP‐drug conjugates allowed for the intratumoral drug release.^227^, ^228^ It is showed that the most effective strategy to enrich the ELP NPs’ tumor accumulation was to thermally cycle the tumors between 37 and 42°C, where NPs aggregated in the vasculature of tumors heated to 42°C and the aggregation reverted and extravasated into tumor tissues as the temperature decreased to 37°C.^226^, ^229^

### 3.5 Switching NP physicochemical properties for enhanced tumor penetration

#### 3.5.1 Size

**Figure 7:**
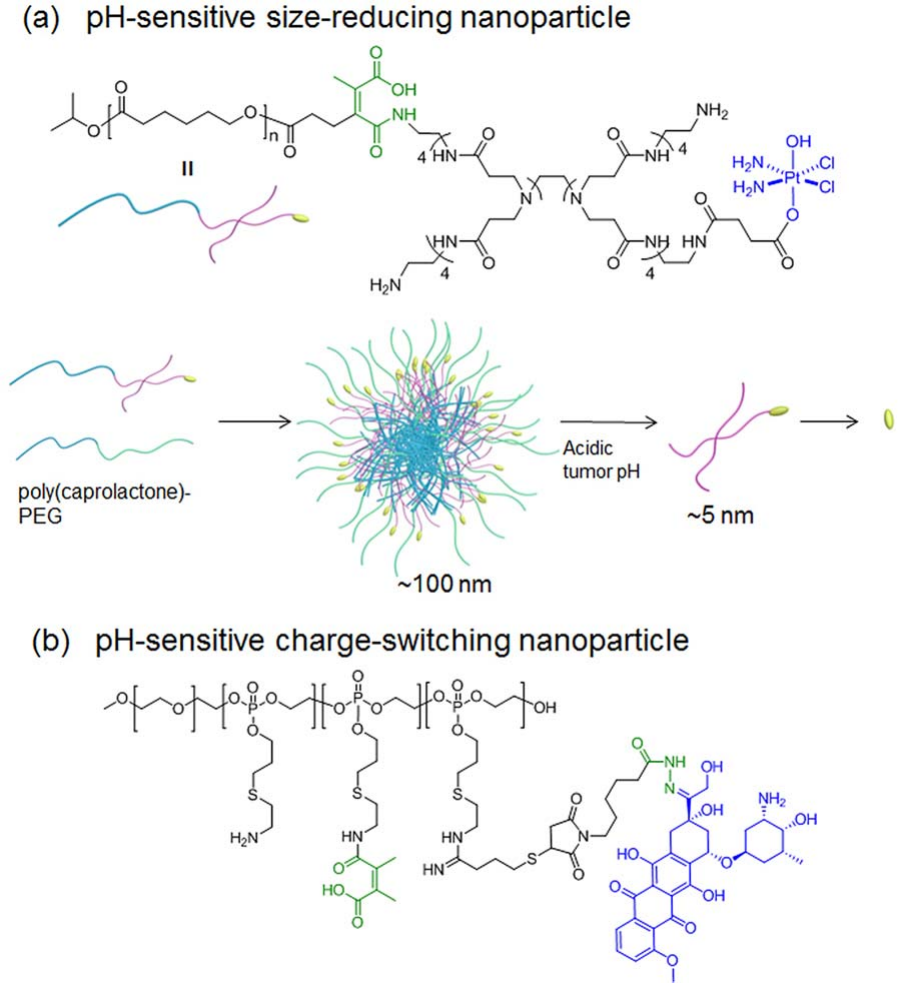
Two examples of polymer‐drug conjugates that can change the nanoparticle's (a) size or (b) charge to improve their tumor penetration. The drugs are highlighted in blue, the pH‐sensitive linkers in green

The diffusion of NPs in solid tumor tissue is hindered by many factors including intratumoral dense extracellular matrix such as collagen and hyaluronic acid.^230^, ^231^ Small‐sized NPs could penetrate deeper in tumor tissue, and not cleared from the tumor as rapidly as much smaller molecular drugs.^232^ However, smaller polymeric NPs are often difficult to formulate and may not have the capacity to have high drug loadings. An alternative delivery approach was proposed to use relatively larger NPs with initial size (still sub‐100 nm NPs), but once docking at tumor sites, NPs were switchable to small particles to facilitate tumor penetration.^233^ The stimuli‐responsive NPs that are able to shrink their sizes by responding to enzymes or light exhibited the enhanced tumor penetration of NPs and improved efficacy.^234^, ^235^ Recently, a new pH sensitive NP was prepared by poly(caprolactone)‐*co*‐poly(amidoamine)—platinum prodrug conjugate with a pH sensitive *cis*‐aconityl linker (degrade ∼pH 6.8) between poly(amidoamine) and poly(caprolactone) (Figure 7a). The initial 100 nm sized NPs degrade in acidic tumor interstitial spaces to 5‐nm poly(amidoamine)—drug conjugates with enhanced diffusion capability.

#### 3.5.2 Surface charge

Positively charged NPs often have short circulation half‐life compared with PEGylated or anionic NPs.^236^, ^237^, ^238^, ^239^ However cationic NPs may penetrate tumors deeper than neutral or anionic NPs due to the attractive electrostatic forces between cationic NPs and anionic endothelial glycocalyx.^240^ Positively charged NPs also generally have better cellular uptake than negatively ones.^241^, ^242^ A pH‐sensitive PDC‐based NP was designed to achieve multi‐stage charge changing to improve delivery efficiency: NP's surface charge maintained slightly anionic at pH 7.4; in tumor tissues with pH ∼ 6.8, the pH‐sensitive *cis*‐aconityl group was cleaved from the surface and expose the positive amine groups, which enhanced the tumor penetration and facilitate cellular uptake; the intracellular low pH in endosome and lysosome (∼5.0) could further promote the intracellular Doxo released from the PDCs by the breakage of hydrozone linkers (Figure 7b).^243^

### 3.6 Supramolecular prodrug conjugates

Prodrugs are pharmacologically inactive or less active drug derivatives, aiming to improve the solubility or pharmacokinetics of drugs. There are some lipid‐drug conjugates in Phase I/II clinical trials, such as a docosahexaenoic acid conjugate of paclitaxel (Taxoprexin),^244^ an elaidic acid conjugate of cytarabine (Elacytarabine),^245^ and a cardiolipin conjugate of gemcitabine.^246^ None of them are designed to assemble into nanostructures. Recently, there have been increased efforts to use well‐designed prodrugs, such as lipid‐drug conjugates, or peptide‐drug conjugates to create NP objects. An obvious advantage of these prodrug conjugates is that they have well‐defined chemical structures, similar to those of small‐molecule drugs; while PDCs often have molecular‐weight distributions and/or multiple components in their nanostructures. Therefore, the in vivo studies of the degradation, metabolism, and excretion of these prodrugs are foreseen more straightforward than those of PDCs.

**Figure 8:**
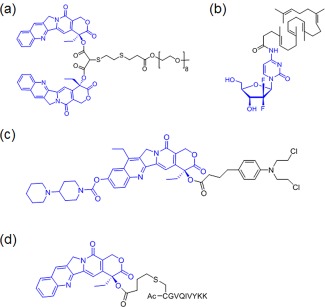
Four representative supramolecular drug conjugates that can assemble into nanoparticle for drug delivery

It is known that amphiphlic or lipid‐like molecules could potentially self‐assemble into supramolecular nanostructures.^247^ Taking advantages of the self‐assembly properties of these small molecules, an amphiphilic prodrug conjugate was synthesized by conjugating two hydrophobic Cpt molecules to a short oligo(ethylene glycol) as the hydrophilic segment via a biodegradable β‐thioester bond. Such amphiphilic prodrug conjugates have high drug loading and form stable 100 nm nanoliposome (Figure 8a).^248^ Similar approach was applied to synthesize amphiphilic PEG‐block‐dendritic polylysine–CPT conjugate that could assemble to nanorod.^249^ Another reported strategy is to conjugate hydrophobic squalene to hydrophilic drugs or prodrugs to construct NPs.^250^ Doxo, gemcitabine and other drugs were “squalenoylated” and formulated into ∼100–150 nm sized NPs (Figure 8b).^251^ An extreme strategy is recently reported to synthesize an amphiphilic drug‐drug conjugate by directly connecting the hydrophilic anticancer drug irinotecan to the hydrophobic anticancer drug chlorambucil via a hydrolyzable ester linker, which can be assembled to NPs with ∼80 nm size (Figure 8c).^252^ Similar conjugate was synthesized between the hydrophilic drug floxuridine with the hydrophobic drug bendamustine.^253^

Besides amphiphilic molecules or lipids, another interesting molecule to prepare prodrug conjugates is the small peptide. It is known that small peptides can assemble into filamentous supramolecular structures.^254^ Such peptide‐drugs conjugates have been intensively studied to formulate hydrogel system, and are reviewed elsewhere.^250^, ^255^, ^256^, ^257^, ^258^ A recent study demonstrate that a rationally designed peptide‐Cpt conjugate be formulated to nanostructures for drug delivery. A β‐sheet‐forming peptide sequence derived from the tau protein was conjugated to Cpt via a redox‐sensitive disulfylbutyrate linker, and the resulting nanostructures could vary from long filaments to short filaments and then to nanotubes with high drug loadings (Figure 8d).^259^, ^260^, ^261^ Studies also showed the choice of the degradable linker between the peptide and Cpt affect the nanostructure. The carbonate linker is more preferred than ester since it minimizes the potential aggregation in cell culture, which could compromise Cpt's potency.^262^

## 4 Outlook

The routine clinical use of PEGylated proteins since 1990s and the recent large investments in ADCs overshadow the development of PDCs. Although so many interesting designs and impressive data presented in this review, there seems a long and arduous journey to bring more PDCs or NPs into clinical practice.^129^, ^263^ Several recent publications try to provide their solutions for the whole nanomedicine field.^264^, ^265^, ^266^ Progress in the field will depend on a fundamental understanding of chemistry, materials science, biology, and clinical practice to allow rational design of optimized NPs of PDCs, tools for delivering them and measure outcomes. In terms of chemistry design, one has to bear in mind the clinical application and whole‐organism pharmacokinetics. One example is the in vivo studies in ADCs revealed an in vitro stable linker may have unexpected instability in vivo and cause reduced efficacy.^267^, ^268^ Many of the first generation PDCs were developed before the concept of nanomedicine and the study of the relationship between NPs’ sizes and their in vivo circulation and intratumoral accumulation; thus such PDCs have moderate molecular weight and small particle sizes (Table 1), which may result in some of the failure in clinical trials. In addition, there lack standard or optimized preclinical or clinical protocols to evaluate PDCs’ stability, tumor penetration, metabolism, and toxicity.^269^ The development of labeling/imaging technique and nano‐device system may help monitor the in vivo use of PDCs. Of all note that many data obtained in animal models cannot be easily translated into humans; in the frequent‐used subcutaneous tumor xenografts the access of the blood to the tumor interstitium is greater than that in solid tumors in patients.^270^, ^271^ Furthermore, the advances in cancer biology can change the landscape of the field rapidly, as seen in the recent promising therapeutics in cancer immunology.^272^, ^273^ Last but not least, nontrivial optimization and engineering has a bearing on the eventual translation of NPs from preclinical experimental models to daily clinical practice.^274^ The PDC and NP preparation should not require complex multistep processes; the scalability of NPs should not represent a problem in industry; the NP product should be sufficiently stable under storage and easy to use in clinics, that is, no complex administration protocols or regimens.^172^, ^275^ All of these prudent considerations will ensure that the field of PDC‐based NPs reaches its full potential for clinical impact in cancer therapy.

## Conflict of interests

The authors are not aware of any affiliations, memberships, funding, or financial holdings that might be perceived as affecting the objectivity of this review.
